# Relationship of the blood metabolome to subsequent carcass traits at slaughter in feedlot Wagyu crossbred steers

**DOI:** 10.1038/s41598-019-51655-2

**Published:** 2019-10-22

**Authors:** Samantha Connolly, Anthony Dona, Lorna Wilkinson-White, Darren Hamblin, Michael D’Occhio, Luciano A. González

**Affiliations:** 1 Sydney Institute of Agriculture & School of Life and Environmental Sciences, The University of Sydney, Camden, NSW 2570 Australia; 2 Hamblin Pty Ltd, ‘Strathdale’, Blue Mountain, Sarina, QLD 4737 Australia; 3 Kolling Institute of Medical Research, Northern Medical School, The University of Sydney, St Leonard’s, NSW 2065 Australia; 4 Sydney Analytical Core Facility, The University of Sydney, Sydney, NSW 2006 Australia

**Keywords:** Metabolomics, Animal physiology

## Abstract

The aim of the present study was to determine the relationships between the blood metabolome and (1) carcass traits with a focus on intramuscular fat (marbling), and (2) the length of time cattle consumed a high-starch diet in feedlot cattle. Blood samples were obtained from 181 Wagyu-crossbred steers between 300–400 days before slaughter when carcass data was collected. ^1^H NMR spectroscopy identified 35 metabolites with 7 positively associated with marbling (3-hydroxybutyrate, propionate, acetate, creatine, histidine, valine, and isoleucine; *P* ≤ *0.05*). Subcutaneous rump fat thickness was positively associated with glucose, leucine and lipids (P ≤ 0.05) and negatively associated with anserine and arabinose (P ≤ 0.05). Carcass weight and growth rate were negatively associated with 3-hydroxybutyrate (*P* < *0.05*), and growth rate was negatively associated with creatine (P < 0.05) and positively associated with aspartate (*P* < *0.05*). Glucose and arginine showed a significant interaction between marbling and number of days animals consumed a high-starch diet (*P* < *0.05*). Sire was the single variable with the largest effect on the relative concentration of metabolites and carcass and production traits. Blood metabolomics helps understand fat and muscle metabolism, and is associated with genotype, and carcass and production traits in cattle offering potential biomarkers suitable to select animals for management and genetic improvement.

## Introduction

Carcass quality and value in beef cattle are determined primarily by intramuscular fat (IMF; marbling), carcass weight, eye muscle area (EMA; *Longissimus Thoracis et Lumborum*, LTL), and subcutaneous fat thickness at the 12^th^ rib or rump site^1^. These are proxy indicators of carcass composition, meat yield, and quality, and are therefore widely used by livestock industries globally^1,2^. In some markets, marbling is the dominant commercial trait because of the relationship between marbling and sensory and eating quality of beef^3^. Wagyu cattle are renowned for high marbling and this trait is the major factor that determines carcass price together with carcass weight^4^. Phenotypic and genetic selection for marbling is difficult as it can only be accurately measured after slaughter and it has a relatively moderate heritability of 0.38^5^. The ability to identify animals with superior carcass traits early in the production cycle would improve productivity and profitability and enable faster genetic progress.

Synthesis of adipose and muscle tissue in cattle occurs from metabolic precursors such as glucose, propionate, acetate, amino acids and lipids, amongst many others^6^. Different fat deposits preferentially utilize certain metabolites as precursors such as IMF preference for propionate and glucose, and subcutaneous fat (SC) preference for acetate^7^. Therefore, the concentrations of metabolites in the blood of cattle would be expected to be correlated with the mass of IMF and SC. The type of diet consumed has also been associated with the blood metabolome at the time of slaughter in cattle^8^ because diet affects fat and muscle deposition^7^. Beef cattle are often raised on pastures and then inducted into feedlots where high grain diets are fed to increase lipogenesis and growth rate^7^. Therefore, the blood metabolome could also be affected by the length of time animals consume a high-grain diet (days on feed, DOF). Reports are lacking on the relationship between the blood metabolome and fat and muscle tissue mass in cattle, or the effect of the number of days animals consume high grain diets on this relationship.

The present study sought to determine the relationship between the blood metabolome and carcass traits in Wagyu-crossbred steers. ^1^H NMR spectroscopy of plasma was used to measure the relative concentration of metabolites as this technique can measure a wide variety of metabolites, is fast and relatively simple^9^. A significant relationship between carcass traits and the concentration of blood metabolites could potentially lead to the identification of biomarkers to predict those traits or assist with genetic improvement. The hypotheses of the present study were (1) that concentrations of blood metabolites were associated with marbling and other carcass traits in Wagyu-cross steers, and (2) that such association was not affected by the length of time animals were fed a high-energy, grain-based feedlot diet. The blood metabolome of steers was ascertained at 65, 119 and 163 DOF and steers were slaughtered after approximately 400 to 440 DOF.

## Material and Methods

### Animals and experimental design

Three mixed groups of F1 (n = 127), F2 (n = 22), and F3 (n = 32) Wagyu-crossbred steers (initial LW 330 ± 1 kg SEM) were inducted on three separate occasions into a commercial feedlot in southern Queensland, Australia. The genotypes of the females crossed with Wagyu bulls were Angus (n = 16), Brahman (n = 28), Brahman crossbred (n = 26), Jersey (n = 3) and Shorthorn (n = 108). The steers generated for the study were the progeny of 23 sires (Japanese Black Wagyu full blood bulls). Group 1 had 49 steers inducted at day 0 (start of the study); Group 2 had 63 steers inducted at day 44; Group 3 had 69 steers inducted at day 97. Animals were housed in one pen but had entered the feedlot as three groups on different dates with Groups 2 and 3 entering 44 and 97 days, respectively, after Group 1. Animals were fed to allow for *ad libitum* consumption of diets that were changed during the period in the feedlot as shown in Table 1.

**Table 1:** Diet formulation and chemical composition of the four rations fed at different stages in the feedlot to Wagyu crossbred steers.

	Unit	Diet 1	Diet 2	Diet 3	Diet 4
**Ingredient, % as fed** ^**1**^					
Days fed Diet		0 to 6	7 to 11	12 to 323	324 to 450
Steam flaked barley	%	19	25	35	42
Steam flaked wheat	%	19	25	13	23
Grower Supplement	%	5	5	0	2
Finisher Supplement	%	0	0	5	4
Molasses	%	14	10	5	4
Vegetable oil	%	0	1	1	1
Brewers sweet grain	%	0	0	19	10
Sunflower Meal	%	9	6	2	0
Corn Silage	%	12	13	15	10
Barley Straw	%	12	11	6	5
Cereal Hay	%	12	4	0	0
**Chemical composition**					
Crude Protein	% DM	13.58	13.56	13.93	13.51
Neutral Detergent Fibre	% DM	31.05	26.53	25.18	20.96
Net Energy of Gain	MJ/kg DM	4.14	4.81	5.23	5.65
Net Energy of Maintenance	MJ/kg DM	6.74	7.49	7.99	8.45
Metabolizable Energy	MJ/kg DM	10.46	11.33	11.85	12.42
Ionophore	ppm	21.13	22.26	22.08	22.45

**Figure 1:**
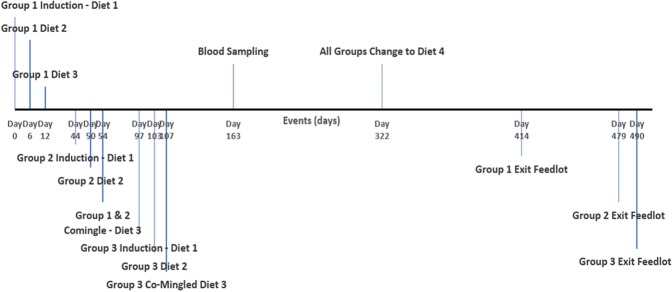
Experimental timeline of events illustrating feedlot entry, diet changes, blood sampling and feedlot exit of Wagyu crossbred steers in relation to experimental day.

Feeding changes, blood sampling and time of slaughter are shown in Fig. 1. Each group of steers was fed diets 1 and 2 for the first 11 days in the feedlot in separate pens then were commingled with other steers in the study. Groups 2 and 3 spent less time on diet 3 compared to Group 1 as they were commingled on different days. Blood samples for metabolome analysis were taken on the same day for all animals which meant that the number of days each group was on diet 3 was: Group 1 (152 days); Group 2 (108 days); Group 3 (54 days).

On the day of blood sampling, animals were removed from their pen at 0600 h before feed distribution and samples were taken between 0700 and 1030 h. Blood was collected from the coccygeal vein using an 18 G needle and evacuated lithium heparin tubes (Vacutainer BD, Becton Dickinson, Franklin Lakes and USA). Samples were immediately placed on ice until centrifugation at 10,000 × g for 15 min. Plasma was stored at −80 °C until analysis. Days in the feedlot at slaughter were 414, 435 and 393 days for Groups 1, 2, and 3, respectively.

The Aus-Meat carcass grading was performed by an accredited assessor at a commercial abattoir^10^. Information recorded included hot standard carcass weight (HSCW), a camera measure of marbling, eye muscle area (EMA), subcutaneous fat depth of the 12^th^ rib, and subcutaneous rump fat thickness at the P8 site. The AUS-meat marbling grading scale ranges from 0 to 9+ with 0 being the lowest and 9+ the greatest marbling. The LTL muscle of each carcass was also examined for percentage marbling using high-quality digital hyperspectral images which is referred to as camera marbling (CM) (HK-333, Hayasaka Rikoh Co. Ltd., Sapporo, Japan)^11^. Marbling score is a subjective and discrete measurement whereas CM is objective and continuous.

### Sample Preparation for metabolome profiling

Sample preparation for metabolic profiling used methods from a published protocol^12^. Samples were thawed at room temperature and an aliquot (350 uL) was mixed with 350 μL of aqueous (80% H_2_O:20% D_2_O) phosphate buffer solution including 0.075 M NaH_2_PO_4_, pH 7.4 (KOH adjusted), 0.1% sodium azide, and 1 mM 3-141 trimethylsilyl-1- [2,2,3,3, -2H4] propionate (TSP) as an internal standard. Samples were vortexed for 30 sec and then centrifuged at 6,000 × g for 10 min. An aliquot of the supernatant (600 uL) for each plasma sample was transferred to 5 mm NMR tubes (Bruker, SampleJet 5 mm, Billerica MA, USA) for ^1^H NMR analysis. A quality control sample comprising equal plasma aliquots of 10 steers was included every 20^th^ sample analysed in the NMR.

Samples were analysed with a Bruker Advance III 600 MHz spectrometer equipped with a 5-mm TCI cryoprobe. Samples were run under automation mode using a Sample Jet with all samples refrigerated at 278 °K until just prior to acquisition. Data were collected at 310 °K for a total of 20 min. ^1^H NMR spectra were acquired using the noesygrrp1d and cpmgpr1d pulse sequences (32 scans collected for each experiment). Irradiation of the solvent (water) resonance is applied during pre-saturation delay (4.0 s) for all spectra and for the noesy also during the mixing time (0.01 s). The pulse sequence parameters, most notably the 90° pulse (~12 μs) are optimised for each sample. The data were collected with approximately 96 k (noesy) or 32 k (cpmg) real data points and processed with an exponential line broadening of 0.3 Hz prior to Fourier transformation.

Data were imported into Matlab 7.0 Software (Matworks, Natick, MA). NMR spectra were aligned and normalised by automatically phasing, baseline correcting and referencing the dataset to the α-C_1_H-Glucose doublet (5.233 ppm)^13^. The residual water (2.42–3.14 ppm) was truncated from the dataset to reduce analytical variability. A PCA was run on the normalised and baseline-corrected spectrums from all samples to visually ensure all the quality control samples clustered together when plotting PC1 vs. PC2 scores. This plot also served to detect any potential outlying datapoint being separated from the main cluster of points. Statistical recoupling of variables was performed on the aligned and normalised spectrum which selected the start and end points of clusters^14^. Therefore, this output contains several clusters or buckets which are chosen as they represent features in the spectral matrix. The cluster value for each sample is simply the area under the curve for each cluster (component or peak). These values are used as relative concentrations and were multiplied by 10^6^ to reduce the number of decimal places. In parallel, the raw spectra were imported into Chenomx® for the assignment of metabolites to these clusters, with metabolites identified using the profiler and library manager models within. This was achieved by comparing ^1^H NMR spectra to the spectral library of Chenomx® NMR Suite Professional (Chenomx Inc., Edmonton, AB, Canada) as well as referencing from published literature and the Livestock Metabolite Database^9,15,16^. Once clusters were assigned to a metabolite, the sum of the area under the curve for all clusters belonging to a metabolite was calculated.

### Statistical analyses

A general linear model was used to analyse the fixed effect of days in the feedlot (days on feed, DOF), breed, generation (F1, F2 or F3), and sire (bull) on carcass and performance traits including Aus-Meat marble, CM, rib fat, P8 fat and HSCW, amongst others. Least Square Means were calculated for each DOF and differences between means adjusted for multiple comparisons using the Tukey method. The same general linear model was used to perform analysis of covariance adding each carcass trait as a covariate to determine their association with the relative concentration of metabolites (dependent variable). This model allowed estimating partial correlation coefficients between carcass traits and the relative metabolite concentration. The model also included the covariate × DOF interaction to test the hypothesis that the relationship (slope) remains constant across DOF. All statistical analyses were done using SAS 9.4 (SAS Institute Inc., Cary, New Jersey, USA). Significant statistical differences were declared at *P* ≤ *0.05* and tendencies discussed at *0.05* < *P* ≤ *0.10*.

Principal component analysis (PCA) were conducted using the relative concentration of the 35 identified metabolites as variables in the model with all PC possible. Then, only those PC with eigenvalues > 1 were selected for the final PCA. Finally, PC1 and PC2 score plots were obtained and datapoints coloured to visualise potential clustering of animals according to DOF.

## Results

Table 2 shows the mean values for carcass and production traits for each DOF group of animals. The DOF groups at the time of blood sampling affected all variables except marbling and Wagyu percentage (P > 0.05; Table 2). Group 1 sampled at 163 DOF showed thinner rump fat, thicker rib fat, and were older compared to animals sampled at 65 (Group 3) and 119 DOF (Group 2) (P < 0.05). Group 2 (119 DOF) had heavier carcasses and live weight compared to Group 3 (65 DOF) (P < 0.05). Sire was the most important fixed effect affecting most carcass and production traits *(P* < *0.05*) except rib fat and EMA (P > 0.10). The female breed only affected age and Wagyu percentage (*P* < *0.05*). Generation (F1, F2, F3) only affected growth rate (*P* < *0.009*) and Wagyu percentage (*P* < *0.001*) but did not affect other carcass traits (*P* > *0.05*).

**Table 2:** Carcass traits, age and live weight of Wagyu cross steers that were blood sampled for metabolomics analysis at different days on feed.

	Days on Feed			P-Value			
	65	119	163	DOF	Breed	Generation	Sire
No. Animals	69	63	49	—	—	—	—
Aus-Meat Marble Score	6.2 ± 0.56	5.8 ± 0.52	5.9 ± 0.52	0.279	0.404	0.384	0.002
Camera marbling (%)	25.8 ± 2.22	25.4 ± 2.07	24.0 ± 2.07	0.182	0.996	0.599	0.008
Rump Fat (mm)	27.6 ± 1.06^A^	27.2 ± 0.97^A^	23.7 ± 1.01^B^	0.006	0.628	0.520	<0.001
Rib fat (mm)	11.2 ± 0.91^A^	10.8 ± 0.84^A^	15.8 ± 0.86^B^	<0.001	0.799	0.519	0.516
Eye muscle area (cm^2^)	40.7 ± 0.89^A^	36.7 ± 0.83^B^	41.4 ± 0.85^A^	<0.001	0.567	0.998	0.308
Growth rate (kg/d)	0.99 ± 0.031^AB^	1.00 ± 0.030^B^	1.05 ± 0.032^A^	0.009	0.097	0.009	<0.001
Carcass weight (kg)	428 ± 5.4^B^	449 ± 6.0^A^	434 ± 5.2^AB^	0.007	0.784	0.755	0.044
Age at Induction (days)	688 ± 29.9^A^	666 ± 28.0^A^	721 ± 28.0^B^	0.001	0.021	0.066	<0.001
Age at Slaughter (days)	1111 ± 18.1^A^	1112 ± 17.2^A^	1147 ± 18.9^B^	0.026	0.030	0.065	<0.001
Induction Live Weight (kg)	326 ± 3.1^A^	336 ± 2.8^B^	321 ± 2.9^A^	<0.001	0.373	0.310	0.028
Exit Live Weight (kg)	745 ± 9.0^B^	783 ± 8.3^A^	766 ± 8.6^AB^	0.003	0.816	0.402	0.054
Wagyu content (%)	72.6 ± 0.76	73.2 ± 0.71	73.0 ± 0.71	0.254	0.004	<0.001	0.002

^A,B,C^Means within rows without a common superscript differ (*P* < *0.05*).

**Figure 2:**
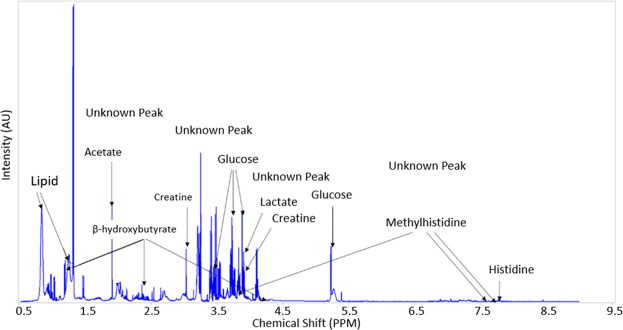
Representative ^1^H-NMR spectrum of plasma from a Wagyu crossbred steer showing clusters assigned to different metabolites (AU: arbitrary units).

The standard recoupling of variables identified 315 features or peaks from the ^1^H NMR spectrum. From these clusters, 35 metabolites were identified using the available Chenomx® database of compounds, and their identity validated from previous literature and the livestock metabolome database. A representative ^1^H NMR spectrum indicating 11 identified metabolites is shown in Fig. 2 to illustrate the multiple features. Also noted are unknown peaks that could not be identified and data on these are not presented in this report.

Table 3 shows descriptive statistics for each metabolite. Glucose had the largest number of clusters due to the large size of the molecule with the greatest area under the curve; however, glucose showed low variability (CV) amongst animals. Smaller metabolites such as formate and acetate had lower number of features, lower area under the curve (relative concentrations) and larger variability across animals.

**Table 3:** Descriptive statistics of the relative concentration of metabolites identified in blood plasma of Wagyu cross steers using H NMR (N = 181).

Variable	No. Clusters	Minimum	Mean	Maximum	Std Error	CV
3-hydroxybutyrate	2	108.9	174.0	284.6	2.30	17.9
Acetate	1	65.6	197.9	371.2	4.40	30.1
Acetone	1	17.0	25.3	49.2	0.34	18.2
Anserine	1	280.6	339.5	336.3	1.97	7.8
Arabinose	1	51.8	89.4	120.2	0.97	14.6
Arginine	1	307.8	387.2	484.4	2.46	8.6
Aspartate	1	17.5	24.4	30.6	0.19	10.7
Carnosine	1	63.7	87.7	124.5	0.69	10.6
Choline	1	63.7	87.6	124.5	0.69	10.6
Citrate	2	47.0	86.0	114.4	0.87	13.7
Citrulline	1	13.1	19.5	19.5	0.17	11.5
Creatine	2	136.2	174.5	221.6	1.14	8.8
Creatinine	2	158.0	238.1	320.7	1.86	10.6
Dimethyl sulfone	1	24.2	35.0	46.9	0.32	12.3
Formate	1	1.91	3.8	32.9	0.17	60.2
Glucose	16	2622	3248	4005	19.23	8
Glutamate	1	16.3	24.2	35.0	0.21	11.9
Glutamine	8	157.7	218.6	280.9	1.40	9.0
Glycine	1	98.7	138.6	200.9	1.39	13.6
Histidine	2	246.0	351.7	490.4	3.52	13.5
Isobutyrate	3	201.9	246.2	301.2	1.20	6.6
Isoleucine	2	116.1	145.2	176.0	0.83	7.7
Lactate	2	323.0	649.8	1484.4	13.88	28.9
Leucine	2	127.9	168.0	209.5	1.06	8.5
Lipid	12	1378.4	1968.5	2409.3	12.85	8.8
Mannose	1	7.8	11.0	17.3	0.12	14.9
Methionine	3	118.6	168.2	215.5	1.11	9.0
Methylamine	1	11.5	36.6	54.3	0.52	19.2
Methyl histidine	3	165.4	200.2	241.6	1.05	7.1
Phenylalanine	3	24.3	34.4	43.82	0.25	9.8
Proline	5	39.7	58.6	101.1	0.64	14.8
Propionate	1	8.3	18.1	24.61	0.15	11.1
Serine	3	126.9	176.0	297.58	1.49	11.4
Tyrosine	3	155.4	191.6	229.83	1.11	7.8
Valine	4	284.4	381.9	480.16	2.36	8.4

**Figure 3:**
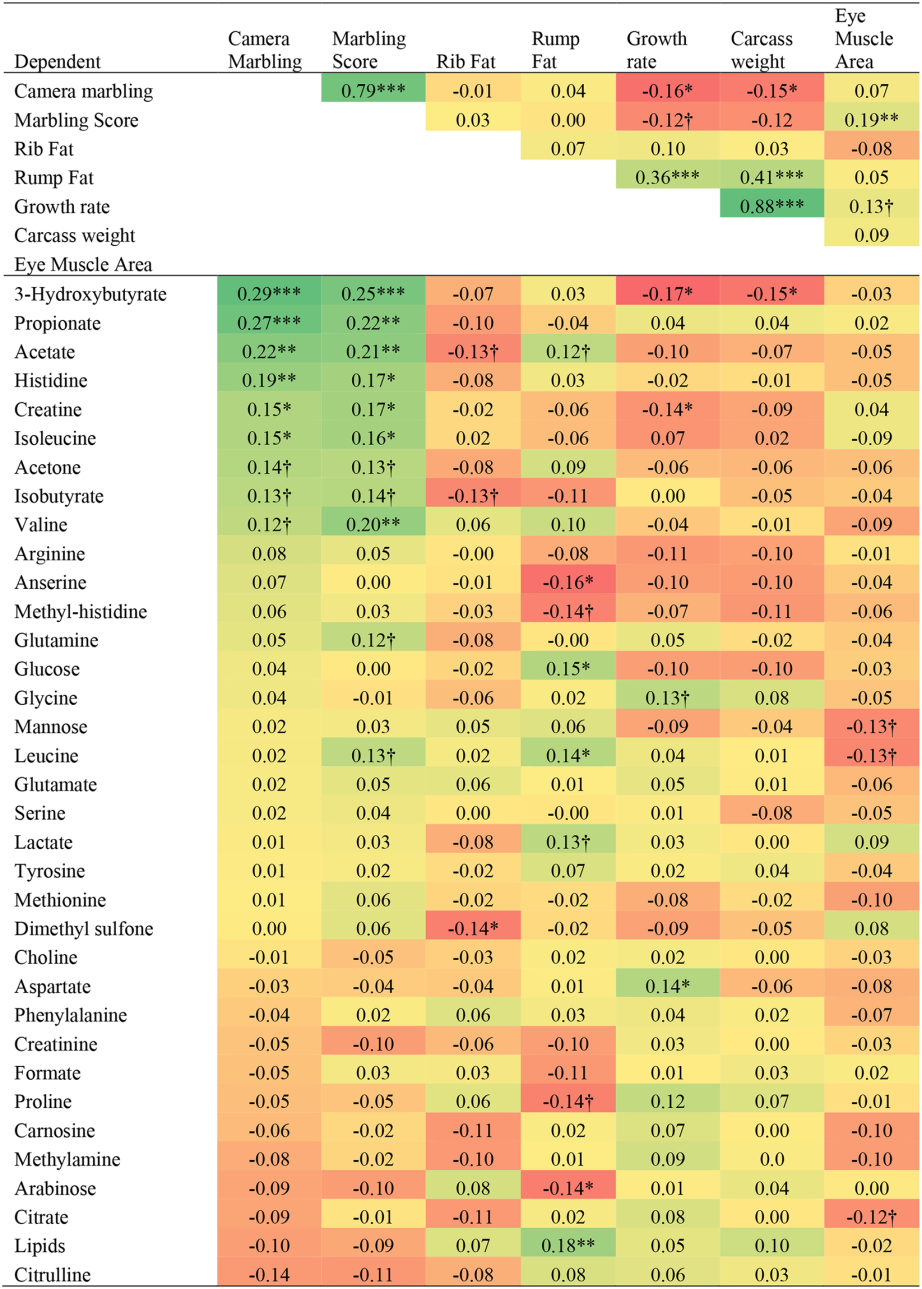
Heat map illustrating partial correlation coefficients of the relationship between metabolites and carcass traits. ***, **, *, † is for P ≤ 0.001, P ≤ 0.01, P ≤ 0.05 and P ≤ 0.10, respectively.

The general linear models indicated that the relative concentration of metabolites was not affected by breed, generation, or the interactions between fixed effects (P > 0.05), and these factors were therefore excluded from the models (data not shown). Results from the analysis of covariance showing partial correlation coefficients between carcass traits and the relative concentration of metabolites are presented in Fig. 3. A strong positive correlation was found between CM and marbling score, and growth rate and carcass weight (*P* < *0.001*). A modest positive correlation was found between growth rate or carcass weight and subcutaneous rump fat (P < 0.001), and a weak negative correlation between marbling and growth rate or carcass weight (*P* < *0.05*). Marbling was not correlated with subcutaneous rump or rib fat (P > 0.10) and a positive correlation was found between marbling score and EMA (P < 0.01; Table 3). Eye muscle area tended to be correlated with growth rate (*P* < *0.10*) but not with other carcass traits (P > 0.10).

Camera marbling was positively correlated with 3-hydroxybutyrate, propionate, acetate, histidine, creatine and isoleucine *(P* ≤ *0.05*; Fig. 3). Similar positive correlations were found between Aus-Meat marble score and the relative concentration of those metabolites although valine also reached significance *(P* < *0.05*). No negative correlations were found between CM or marble score and the relative concentration of metabolites (*P* > *0.05*). Subcutaneous rib fat showed a negative correlation with dimethyl sulfone (P < 0.05) and a negative tendency with acetate and isobutyrate (P < 0.10). Subcutaneous rump fat depth was positively correlated with lipids, glucose and leucine (*P* < *0.05*), and tended to be positively correlated with acetate and lactate (P < 0.10). Rump fat was negatively correlated with anserine and arabinose (*P* < *0.05*; Fig. 3). Carcass weight, growth rate and eye muscle area did not show positive correlations with metabolites (*P* > *0.05*) except for that between aspartate and growth rate (P < 0.05). Carcass weight and growth rate were negatively correlated with 3-hydroxybutyrate, and growth rate also with creatine (*P* < *0.05*). Eye muscle area did not show significant correlations with any metabolite except for a negative trend with mannose, leucine and citrate (P ≤ 0.10; Fig. 3).

**Figure 4:**
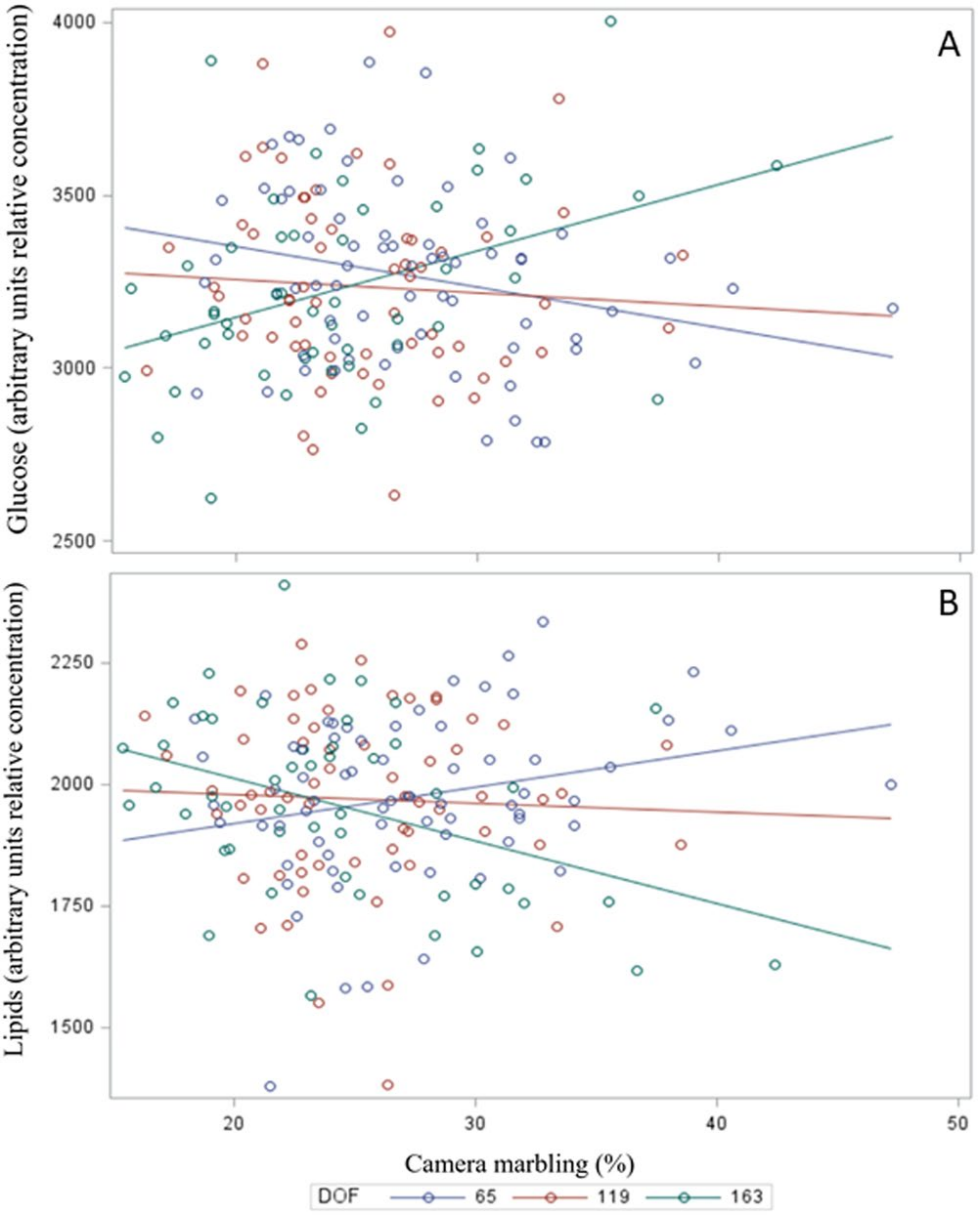
Analysis of covariance for the relative concentration of glucose and lipids in plasma of feedlot Wagyu crossbred steers showing the DOF and CM interaction.

The analysis of covariance for CM showed that sire affected the relative concentration of 16 metabolites (P ≤ 0.05) and tended to affect another 6 metabolites (P ≤ 0.05; Table 4). Confirming results from correlation analysis, the relative concentration of 3-hydroxybutyrate, propionate, acetate, creatine, and histidine increased with CM (P < 0.05) and no metabolites decreased with CM. The main effect of DOF affected anserine, arginine, glucose and methyl histidine (P ≤ 0.05); however, these metabolites also showed a DOF × CM interaction as it was the trend observed for lipids as well (P ≤ 0.10; Table 4). Figure 4 illustrates the linear relationship between the relative concentration of glucose and marbling for each DOF group. Glucose showed a linear decrease with marbling at 65 DOF *(P* < *0.05*), no effect at 119 DOF (*P* > *0.10*) and increased with marbling at 163 DOF (*P* < *0.05*). In contrast, the relative concentration of arginine (data not shown) and lipids (Fig. 4) increased with marbling at 65 DOF (*P* < *0.05*), no change at 119 DOF (*P* > *0.05*) and decreased at 163 DOF (*P* < *0.05*).

**Table 4:** Effect of days on feed (DOF) and camera marbling on the relative concentration of blood metabolites in Japanese Black Wagyu crossbred steers.

Metabolite	Days on Feed			Marbling	P-value			
	65	119	163	Regression ± SE	DOF	Marbling	Marb × DOF	Sire
3-Hydroxybutyrate	170.4 ± 5.46	181.0 ± 4.96	180.4 ± 5.15	1.82 ± 0.504	0.488	<0.001	0.249	0.482
Acetate	202.3 ± 10.61	211.9 ± 9.62	181.9 ± 10.01	3.04 ± 0.985	0.156	0.003	0.159	0.529
Acetone	24.1 ± 0.83	25.6 ± 0.75	26.6 ± 0.78	0.1 ± 0.077	0.668	0.055	0.354	0.657
Anserine	347.1 ± 4.51^A^	335.1 ± 4.09^B^	335.8 ± 4.26^B^	0.43 ± 0.426	0.005	0.332	0.014	0.062
Arabinose	91.7 ± 2.18	90.6 ± 1.98	87.7 ± 2.06	−0.17 ± 0.2	0.575	0.207	0.353	0.008
Arginine	393.2 ± 5.76^A^	379.5 ± 5.22^B^	390.9 ± 5.43^AB^	0.51 ± 0.538	0.032	0.240	0.026	0.071
Aspartate	24.9 ± 0.46	24.6 ± 0.42	24.0 ± 0.43	0.03 ± 0.042	0.401	0.685	0.350	0.303
Carnosine	48.2 ± 1.3	50.6 ± 1.18	47.6 ± 1.22	0.12 ± 0.119	0.779	0.424	0.642	0.093
Choline	88.1 ± 1.59	87.4 ± 1.44	85.6 ± 1.5	0.05 ± 0.144	0.887	0.937	0.747	0.023
Citrate	86.4 ± 2.08	88.6 ± 1.89	84.1 ± 1.96	0.29 ± 0.19	0.551	0.219	0.359	0.126
Citrulline	19.0 ± 0.4	19.2 ± 0.36	19.5 ± 0.37	−0.06 ± 0.036	0.117	0.153	0.150	0.024
Creatine	387.7 ± 6.5	388.9 ± 5.9	376.1 ± 6.14	1.49 ± 0.594	0.499	0.033	0.721	0.012
Creatinine	31.1 ± 0.66	32.7 ± 0.6	33.7 ± 0.62	−0.08 ± 0.062	0.829	0.483	0.903	0.012
Dimethyl sulfone	34.5 ± 0.76	34.7 ± 0.69	33.5 ± 0.71	0.01 ± 0.069	0.915	0.952	0.962	0.028
Formate	3.67 ± 0.12	3.62 ± 0.11	3.49 ± 0.11	0.01 ± 0.011	0.775	0.501	0.600	0.116
Glucose	3,263 ± 43.8^A^	3,182 ± 39.69^B^	3,240 ± 41.29^A^	1.22 ± 4.059	0.033	0.599	0.031	0.007
Glutamate	25.0 ± 0.48	25.3 ± 0.43	23.6 ± 0.45	0.04 ± 0.045	0.430	0.760	0.749	0.000
Glutamine	219.9 ± 3.37	221.4 ± 3.06	212.4 ± 3.18	0.37 ± 0.31	0.952	0.441	0.977	0.006
Glycine	145.9 ± 3.19	137.1 ± 2.89	131.6 ± 3.01	0.09 ± 0.304	0.484	0.628	0.490	0.285
Histidine	346.4 ± 8.22	356.4 ± 7.46	361.2 ± 7.76	1.8 ± 0.751	0.609	0.009	0.462	0.035
Isobutyrate	243.2 ± 2.83	247.3 ± 2.57	246.2 ± 2.67	0.44 ± 0.257	0.963	0.068	0.893	0.011
Isoleucine	148.6 ± 1.99	143.4 ± 1.8	139.0 ± 1.88	0.38 ± 0.189	0.657	0.186	0.973	0.992
Lactate	620.4 ± 31.88	657.3 ± 28.92	748.0 ± 30.08	−1.24 ± 2.984	0.881	0.854	0.868	0.064
Leucine	166.7 ± 2.51	167.6 ± 2.27	163.9 ± 2.37	0.1 ± 0.227	0.946	0.768	0.929	0.076
Lipid	1,989 ± 28.7	1,977 ± 26.04	1,931 ± 27.08	−2.15 ± 2.66	0.185	0.167	0.073	0.014
Mannose	10.9 ± 0.29	11.3 ± 0.26	11.0 ± 0.27	−0.01 ± 0.026	0.897	0.749	0.970	0.046
Methionine	171.0 ± 2.57	171.1 ± 2.33	162.2 ± 2.42	0.16 ± 0.24	0.758	0.897	0.998	0.012
Methylamine	36.4 ± 1.25	38.3 ± 1.14	35.7 ± 1.18	0.15 ± 0.115	0.707	0.287	0.533	0.210
Methyl histidine	204.0 ± 2.47^A^	198.8 ± 2.24^B^	197.5 ± 2.33^B^	0.23 ± 0.23	0.040	0.404	0.096	0.096
Phenylalanine	33.9 ± 0.63	34.2 ± 0.57	33.9 ± 0.59	−0.03 ± 0.056	0.946	0.612	0.915	0.618
Proline	58.7 ± 1.55	57.7 ± 1.4	57.4 ± 1.46	−0.08 ± 0.14	0.968	0.472	0.910	0.204
Propionate	17.2 ± 0.32	17.9 ± 0.29	18.6 ± 0.31	0.1 ± 0.031	0.904	<0.001	0.899	0.004
Serine	174.5 ± 3.26	172.7 ± 2.95	172.6 ± 3.07	−0.09 ± 0.294	0.786	0.791	0.837	0.260
Tyrosine	192.5 ± 2.49	192.9 ± 2.26	190.3 ± 2.35	0.04 ± 0.226	0.604	0.864	0.599	0.000
Valine	375.1 ± 5.65	380.4 ± 5.13	374.7 ± 5.33	0.84 ± 0.511	0.936	0.104	0.882	0.109

^A,B,C^Means without a common superscript differ (*P* < *0.05*).

**Figure 5:**
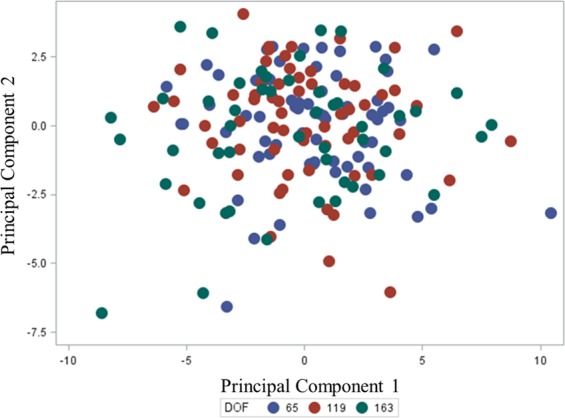
Principal component analysis of 35 blood metabolites of Wagyu crossbred steers showing PC 1 vs PC 2 with the days on feed (DOF) group coloured for each data point.

The PC1 explained 11.61% of the variability within the dataset of 35 identified metabolites and PC2 explained 4.46% of the variability. The score plot with both PC1 and PC2 demonstrated that there was no clustering of animals according to DOF with the data points from different groups randomly distributed (Fig. 5).

## Discussion

A hypothesis tested in the present study was that the concentrations of blood metabolites were associated with marbling and other carcass traits in Wagyu crossbred steers. This hypothesis was supported through significant associations between the relative concentration of metabolites and carcass traits. The blood metabolome was ascertained at approximately 300 days before steers were slaughtered to collect data on carcass traits. This is highly important because it suggests that the metabolome could be used for the early identification of steers with propensity to marble, which could have major implications for efficient utilization of feed in steers that produce a carcass of high value.

### Effects of genotype on carcass traits and the metabolome

The potential of metabolomics to inform genetic selection is supported by the significant effect of sire on both carcass traits and the metabolic profile of animals. Sire was the single most important factor affecting carcass and performance traits, and the relative concentration of metabolites. Sixteen of 35 metabolites were affected, and a further 6 metabolites tended to be affected, by sire. On average, sire explained 21.5% and marbling 4.8% of the variability in the relative concentration of these significant metabolites. The effect of sire on carcass traits is expected because of the known heritability of these in Wagyu cattle^3^. However, the effect of genotype on blood metabolomics of cattle has not previously been reported to the authors’ best knowledge. These results suggest that genetic progress could be improved considering metabolomic information together with pedigree and genomic information as previously suggested^17,18^.

### Metabolomics to improve the understanding of fat and muscle biology

A recent review found 79 articles that identified 8 or more metabolites in cattle^9^, suggesting that the use of metabolomics in bovine studies is relatively unexplored. The present study found positive and negative associations between multiple metabolites and the extent of tissue accrued in different depots (intramuscular and subcutaneous fat, growth rate, eye muscle area and carcass weight at slaughter). This suggests that blood metabolomics in cattle could help to unravel metabolic pathways and mechanisms of fat and muscle in body systems. Previous studies reported that the relationship between biomarkers and phenotypes is not robust and depends on the trait^19^. It was concluded that whilst blood proteomics has potential for biomarkers of tenderness in cattle it is unlikely that a single biomarker will have an outstanding effect^19^. In agreement with this, none of the metabolites in the present study were strongly correlated with carcass traits. However, the fact that several metabolites showed significant correlations with carcass traits indicates that further research is warranted on the identification and potential application of multiple biomarkers or metabolites.

Japanese Black Wagyu cattle may be a good model to study fat metabolism due to the extent of marbling in this breed. Marble scores ranged from 3 to 9+ in the present study (average of 5.97) and such variability across animals decreases with increased Wagyu content^20^. However, both carcass weight and marbling should be considered together in any balanced breeding program because the value of a carcass is determined by both traits and there is often a trade-off with a negative correlation between these two traits^21^. The present study partly agrees with this observation because animals that grew faster tended to have larger EMA, heavier carcasses, and lesser marbling but thicker subcutaneous rump fat. Some metabolites reflected this negative association such as 3-hydroxybutyrate and creatine which were positively associated with marbling and negatively with growth rate or carcass weight. Both 3-hydroxybutyrate and creatine are key energy sources for cattle; however, 3-hydroxybutyrate is a key metabolite involved in fat tissue metabolism^22^ whereas creatine is a key metabolite involved in muscle and brain tissue metabolism facilitating the recycling of ATP^23^. In agreement with the present study, double-muscled Belgian Blue cattle had lower plasma concentrations of creatine, heavier carcasses and lower proportion of fat tissue compared to conventional Belgian Blue cattle^23^. A previous study^24^ reported higher concentration of 3-hydroxybutyrate for genetic lines with lighter carcasses and lower body fat proportion in Charolais x Holstein crosses although marbling score was not different between lines. The metabolite 3-hydroxybutyrate originates from either absorption of acetate from the rumen (~70%) or hepatic oxidation of long chain fatty acids, particularly from fat mobilization during negative energy balance^6^. Animals in the present study were growing and in positive energy balance. Therefore, circulating 3-hydroxybutyrate in cattle seems to reflect different metabolic pathways depending on whether animals are in positive or negative energy balance.

Most of the energy used by ruminants comes from ruminal microbial degradation of feed which produces volatile fatty acids (VFA) with acetic, propionic and butyric acids being the most important^25^. Acetate is a key lipogenic substrate in ruminants and once absorbed in the blood most of the acetate is converted to 3-hydroxybutyrate, oxidized via the tricarboxylic acid cycle (TCA) or used for fatty acid synthesis^26^. Propionate reaches the liver where it is either oxidized or enters the TCA cycle as succinyl-CoA to form glucose. However, there is no apparent agreement in the literature as to which metabolites are the most important precursors of the different fat depots and muscle defining body composition in cattle. It has been reported that acetate and glucose are the major precursors for fatty acid biosynthesis, with glucose being preferred by intramuscular adipocytes and acetate by subcutaneous fat depots^6,27–29^. It has also been shown that plasma propionate increases the secretion of insulin which activates lipogenic enzymes and accelerates fatty acid synthesis increasing intramuscular fat^30^. A previous review^6^ also concluded that the main precursors for IMF deposition in ruminants are lactate and glucose, and acetate to a lower extent. In contrast, we did not find consistent relationships between lactate or glucose and marbling to support previous observations. The present study suggests that both circulating propionate and acetate have a similar positive influence on marbling however 3-hydroxybutyrate seems to play the most important role.

Acetate was the only metabolite that tended to be positively correlated with both intramuscular and subcutaneous rump fat depots, and negatively with subcutaneous rib fat thickness. In addition, subcutaneous rump fat tended to be positively associated with glucose, lipids, leucine and lactate. However, none of these metabolites were correlated with marbling, subcutaneous rib fat, carcass weight or growth rate. The positive correlation between lipids and rump fat contrasts with trends reported in post-partum dairy cows losing weight under negative energy balance, which showed that circulating lipids (triglycerides, phospholipids and cholesterol) increased concomitantly with a decrease in body weight, condition score, backfat thickness and LTL muscle diameter^31^. Results from the present study seem to challenge previous studies with respect to which precursors have a strong influence on fat accretion in different depots and further research is required in this area.

Valine, isoleucine and leucine are branched chain amino acids known to enhance lipolysis at insufficient or excessive concentrations but can also increase lipogenesis^32^. In the present study, these amino acids were positively associated with marbling and in the case of leucine with rump fat as well. Isobutyrate is a branched chain VFA produced by rumen fermentation of amino acids^33^ however their effect on fat synthesis and deposition in cattle seems unknown. Based on the positive associations between these metabolites and IMF reported in the present study, we speculate that branched chain amino acids, histidine and isobutyrate could promote lipogenesis or fat deposition in IMF, or both.

### Effect of DOF on the blood metabolome

The second objective of the present study was to investigate the influence of the length of time that cattle consumed a high grain and starch diet (DOF) on the blood metabolome, and to determine if the correlation between metabolites and marbling was affected by DOF. The DOF at sampling may need to be considered for practical application if this influences the metabolomic profile of animals. Both carcass and production traits were affected by DOF, which was unexpected because animals in the present study were randomly selected from a commercial producer of feeder cattle from the same breeding cohort. Importantly, marbling was not different between DOF groups, indicating that the results reported on the relationship between the metabolome and marbling is not confounded by DOF groups having different marbling. No metabolites were affected by the main factor of DOF and, therefore, the relative concentration of metabolites does not seem to be affected by the length of time animals consume a high grain diet in the ranges evaluated in the present study (i.e. early in the feedlotting process). These results were supported by the PCA which did not show any clustering of data points according to DOF.

Arginine, glucose and lipids were the only metabolites influenced by the interaction between DOF and CM. The relative concentration of glucose increased, and lipids decreased, as marbling increased in animals sampled later (163 DOF); however, the opposite trend was reported for animals sampled earlier in the feeding period (65 DOF). These findings suggested that DOF needs to be considered to predict marbling from arginine, glucose and lipids. However, the most promising metabolites to predict marbling such as 3-hydroxybutyrate, propionate and acetate were not affected by the length of time animals consumed a high grain diet in the feedlot.

A similar study^34^ to the present one, identified 45 metabolites and examined the relationship between the blood metabolome and residual feed intake of feedlot cattle sampled at 14, 42 and 70 DOF. The metabolites selected as predictors of residual feed intake differed amongst DOF; however, it was unclear if these results were related to recent diet changes, the length of time animals consumed a high-grain diet, or different environmental conditions across sampling dates, amongst others. The present study used animals well adapted to the high-grain diet (animals were consuming the high grain diet for at least 54 days at time of sampling) and all animals were sampled on the same date to avoid the effect of environmental conditions.

## Conclusion

Blood metabolomics in cattle shows potential biomarkers that could help to better understand fat and muscle metabolism and predict economically important carcass traits at 10 to 14 months before slaughter. These could be used to identify and select individual animals with desirable carcass traits. The length of time in the feedlot when animals are sampled appears not to be a critical factor affecting the blood metabolome. Genotype has a large influence on both blood metabolomics and carcass and production traits suggesting that ^1^H NMR metabolomics could assist with genetic improvement of cattle for relevant production and carcass traits and meat quality.

### Animal ethics

The study had animal ethics approval from The University of Sydney Animal Ethics Committee: Protocol no. 1125. The study was undertaken in accordance with the Australian code for the care and use of animals for scientific purposes 8th Edition 2013.

## Data Availability

The data and computing programs used in this manuscript may be available from the corresponding author on request and if approved by funding bodies to do so. Restrictions apply to the availability of these data, which were used under license for the current study, and so are not publicly available.
